# Duck Tembusu virus exhibits neurovirulence in BALB/c mice

**DOI:** 10.1186/1743-422X-10-260

**Published:** 2013-08-14

**Authors:** Shuang Li, Xiaoxia Li, Lijiao Zhang, Yongyue Wang, Xiuling Yu, Kegong Tian, Wenliang Su, Bo Han, Jingliang Su

**Affiliations:** 1 Key Laboratory of Animal Epidemiology and Zoonosis, Ministry of Agriculture, College of Veterinary Medicine, China Agricultural University, Beijing 100193, China; 2 Innovation Research Institute, Beijing Vica Group, Beijing 100085, China; 3 Veterinary Diagnostic Laboratory, China Animal Disease Control Center, Beijing 100193, China

**Keywords:** Duck Tembusu virus, Flavivirus, Neurovirulence, BALB/c mice

## Abstract

**Background:**

Duck Tembusu virus is a member of the Ntaya group in the genus *Flavivirus*. The virus has been responsible for severe duck egg-drop syndrome in China since 2010. Its emergence and rapid spread have caused great economic loss for the poultry industry. The epidemiology of the virus infection and the potential threat to public health is of great concern because of the infective and zoonotic nature of flaviviruses.

**Results:**

In this study, the pathogenicity of duck Tembusu virus in BALB/c mice was investigated. Infected mice developed clinical signs, including loss of appetite, ruffled hair, weight loss, disorientation, blindness and paralysis of hind limbs from six days post- infection following intracerebral inoculation. Morbidity was 100%, with mortality ranging from 20 to 80% in three- to eight-week-old mice. High virus titers were recovered from the brain, and the virus was distributed in several organs. Histologically, there was widespread non-suppurative encephalitis in the brain. Lymphocyte depletion in the spleen was observed, along with fatty degeneration in the liver and kidney.

**Conclusions:**

Our results demonstrate, for the first time, that duck Tembusu virus is highly neurovirulent in BALB/c mice. The mouse model used in this work was able to produce Tembusu virus infection and could be useful for elucidating some of the aspects of the pathophysiology of other flavivirus infections.

## Introduction

The genus *Flavivirus* contains viruses transmitted by mosquitoes or ticks and viruses with unknown vectors. More than 70 members have been recognized worldwide. These range from major zoonotic pathogens such as dengue virus (DENV), Japanese encephalitis virus (JEV), yellow fever virus (YFV) and West Nile virus (WNV) to viruses that cause minor human febrile diseases or unknown diseases [1]. Flaviviruses have been shown to have a significant propensity to spread, emerge and establish transmission in a new geographic area if appropriate vectors and vertebrate hosts are present [2,3]. The West Nile virus outbreak that occurred in the USA in 1999 is the best example of this phenomenon to date [4]. Other recent outbreaks of flavivirus infections include the Usutu virus in Europe in 2001 [5] and the Bagaza virus in India and Spain [6,7].

Tembusu virus (TMUV), a member of the Ntaya virus (NTAV) group within the *Flavivirus* genus, was first isolated from mosquitoes in Malaysia in the 1950s [8,9]. In 2000, a subtype of TMUV, the Sitiawan virus, was reported to cause encephalitis and growth retardation in broiler chickens [9]. In 2010, a severe outbreak of a TMUV-related virus, Baiyangdian virus (BYDV), in duck flocks was described in China. BYDV was observed to cause ovary-oviduct disease and declines in feed uptake and egg drop in laying ducks [10]. The virus spread quickly around the major duck-producing regions in China, affecting both ducks and geese of different ages [11,12]. Infected young birds presented various neurological signs to certain degrees, ranging from recumbency to leg and wing paralysis. The virus was also referred to as Tembusu-like virus and duck egg-drop syndrome virus (DEDSV) in China [13]. Although serological evidence suggests that birds may act as reservoirs of TMUV [14], it is difficult to assign a specific reason for the emergence of the disease in China. Mosquito transmission among ducks has been predicted [15], but the epidemiological features of duck TMUV infection remain to be elucidated. Neutralizing antibodies against TMUV have been detected in sera from humans and Bornean orangutans [16], suggesting a preference to infect mammals. Therefore, the potential threat of the virus to public health cannot be overlooked.

The aim of this study was to investigate the virulence of TMUV originating from ducks in BALB/c mice. Virus replication and pathological changes in mice after intracerebral inoculation were assessed.

## Results

### Influence of infection route and mouse age on pathogenicity

Infection of three- to six-week-old mice indicated that mice were susceptible to intracerebral (i.c.) inoculation of duck TMUV, resulting in three to four deaths out of five infected mice at a dose of 7.5 × 10^4^ PFU/mouse in triplicate experiments. All infected mice exhibited significant clinical signs from day 6 after i.c. inoculation, including feed uptake decline, loss of body weight and neurological signs. Some surviving mice displayed significant paralysis in their hind legs. In contrast, subcutaneous (s.c.) and intraperitoneal (i.p.) infection did not cause any signs of illness or death. In addition, seven- and eight-week-old mice suffered one or two deaths out of five mice after intracerebral inoculation, which was fewer than the number of deaths in mice that were three to six weeks old. Infection dose test results indicated that intracerebral inoculation of 7.5× 10^2^ PFU per mouse caused 50% mortality of three-week-old mice, and the same death rate was observed in six-week-old mice inoculated with 1.7 × 10^4^ PFU. The increase of the 50% lethal dosage suggested that the resistance of the mice increased with age.

### Virus replication in the brain and periphery organs of infected mice

To monitor viral replication in different organs of mice, infectious viral titers in tissues were determined 3–12 days after infection of six-week-old BALB/c mice. High titers of virus were isolated from brains on days 3, 6 and 9 post-infection (Table 1). Infectious virus was not detected in the blood, spleen, liver and kidneys of infected mice as shown by plaque assays.

**Table 1:** Infectious viral titers in brains of duck TMUV-infected six-week-old BALB/c mice

**Days post-infection (*n*** **= 5)**	**PFU/mouse** $\left(\bar{X}\pm \mathrm{SD}\right)$
3	6.5 × 10^4^ ± 4.5 × 10^4^
6	5.8 × 10^5^ ± 1.1 × 10^5^
9	7.0 × 10^5^ ± 6.1 × 10^5^
12	0

Because the detection limit of the plaque assay requires a much higher titer of virus, we decided to investigate the distribution of the virus in the spleen, liver and kidneys by means of real-time PCR. Total RNA was extracted from tissue samples at different time-points. Viral RNA was detected in the spleens and livers from all infected mice between days 3 to 9 post-infection (Table 2). Three of five kidneys were positive on day 6 after infection. These observations suggest limited replication of the virus in visceral organs.

**Table 2:** Duck Tembusu virus RNA detection in tissues of six-week-old BALB/c mice tissues sacrificed at various time-points

**Days post-infection**	**Tissue sample**			
	**Brain**	**Spleen**	**Liver**	**Kidney**
3	100% (5/5)	100% (5/5)	100% (5/5)	0% (0/5)
6	100% (5/5)	100% (5/5)	100% (5/5)	60% (3/5)
9	100% (5/5)	100% (5/5)	100% (5/5)	0% (0/5)
12	100% (5/5)	0% (0/5)	0% (0/5)	0% (0/5)

### Histological changes

**Figure 1:**
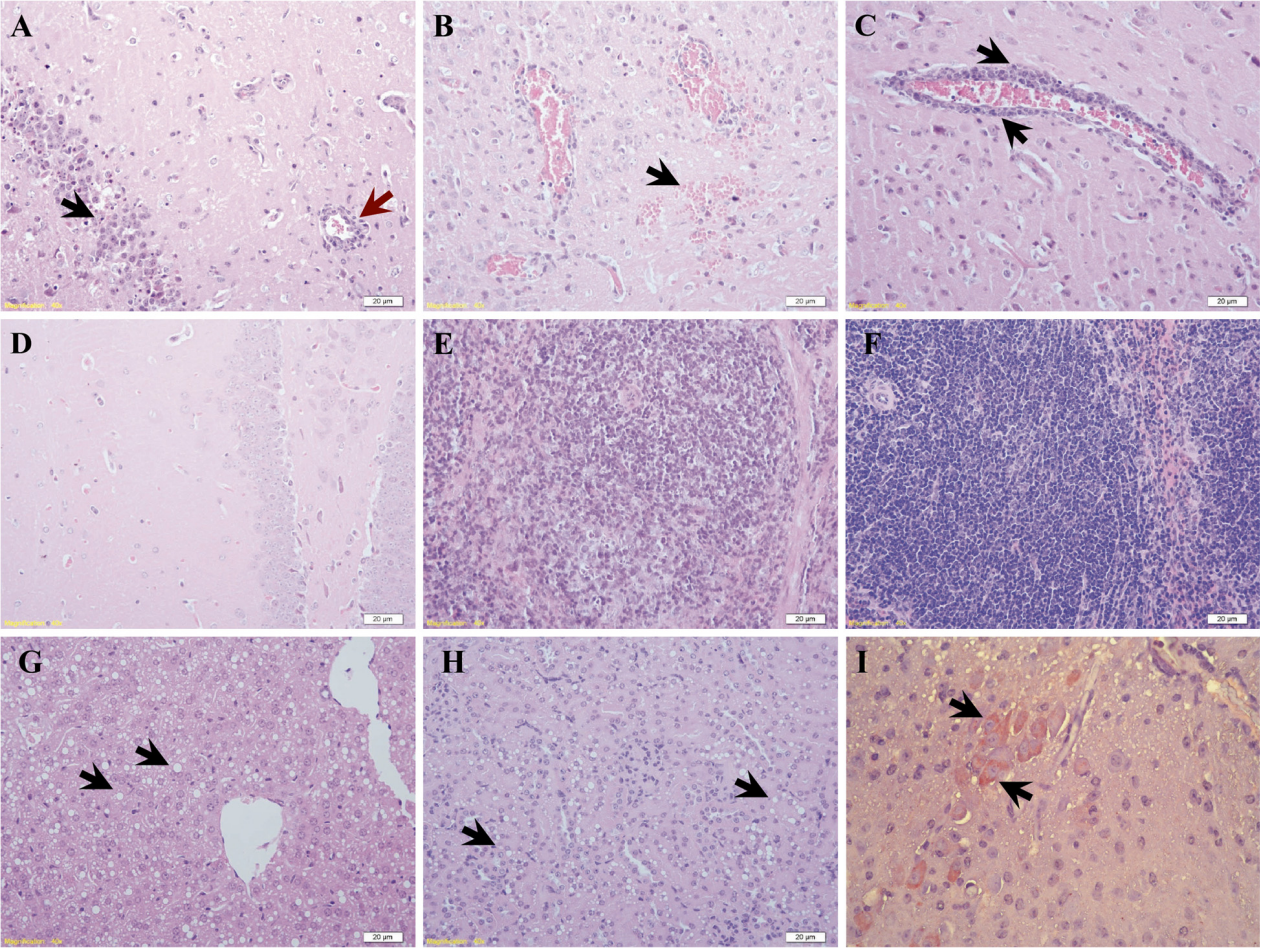
**Histological changes in mice infected with 7.5 × 10**^**3 **^**PFU of duck TMUV *via *the i.c. route. (A-D)** Brain sections from infected mice showing **(A)** focal activation of glial cells (black arrow) and lymphoid perivascular cuffing (red arrow), **(B)** mild hemorrhage (arrow), **(C)** perivascular cuffing formation (arrow) and **(D)** non-infected controls (HE-stained). **(E)** Spleen sections from infected mice showing moderate lymphocyte depletion in the germinal center with the reticular structure preserved of infected mouse. **(F)** Spleen sections in control group. **(G** and **H)** Sections of liver and kidney showing extensive steatosis (arrow). **(I)** Viral antigen was detected in the cytoplasm of neural cells in the brain (arrow). (Scale bar = 20 μm).

We performed histological examination of several tissues from mice sacrificed at three, six, nine and 12 days post-infection (*n* = 3). Changes were observed mainly in the brains of infected mice, with characteristics of encephalitis induced by neurovirulent viruses observed. Inflammatory infiltration was visible in the brain of one infected mouse (1/3) on day 3 post-infection. By day 6, lymphoid perivascular cuffing, microglial activation and mild hemorrhage were also observed, and the maximum level of changes were recorded on day 9 post-infection (Figure 1A-C).

In the spleen, lymphoid depletion was observed in the germinal centers of one infected mouse on day 3 post-infection. By day 9, all three infected mice exhibited moderate depletion of lymphocytes in the germinal centers. The reticulum cells were preserved, and the reticular structure was not destroyed (Figure 1E). Moderate fatty degeneration was observed in the liver and kidneys on day 9 post-infection (Figure 1G and 1H). No apparent microscopic changes were observed in the heart and lungs in this study. The occurrence and histological scales of these changes are summarized in Table 3.

**Table 3:** Histological summary of infected six-week-old BALB/c mice euthanized at various time-points post-inoculation

**Tissues**		**Days post-infection**			
		**3**	**6**	**9**	**12**
Brain	Control	-	-	-	-
	Infected	1/3+	3/3++	3/3#	3/3++
Heart	Control	-	-	-	-
	Infected	-	-	-	-
Lung	Control	-	-	-	-
	Infected	-	-	-	-
Liver	Control	-	-	-	-
	Infected	-	-	1/3++, 2/3#	-
Spleen	Control	-	-	-	-
	Infected	1/3+	3/3+	3/3++	1/3+
Kidney	Control	-	-	-	-
	Infected	-	-	2/3++, 1/3#	-

-, none; +, 1–3 focal inflammatory infiltrates, perivascular cuffing and microglial hyperplasia per high power field (HPF); ++, lesions covering 25% of a HPF; #, lesions covering >75% of a HPF.

Sections of brain, spleen, liver and kidneys were detected by immunohistochemistry as described in the Materials and Methods. Duck TMUV antigen was only observed in neural cells of the brain of the infected mice (Figure 1I).

### Liver function studies

**Figure 2:**
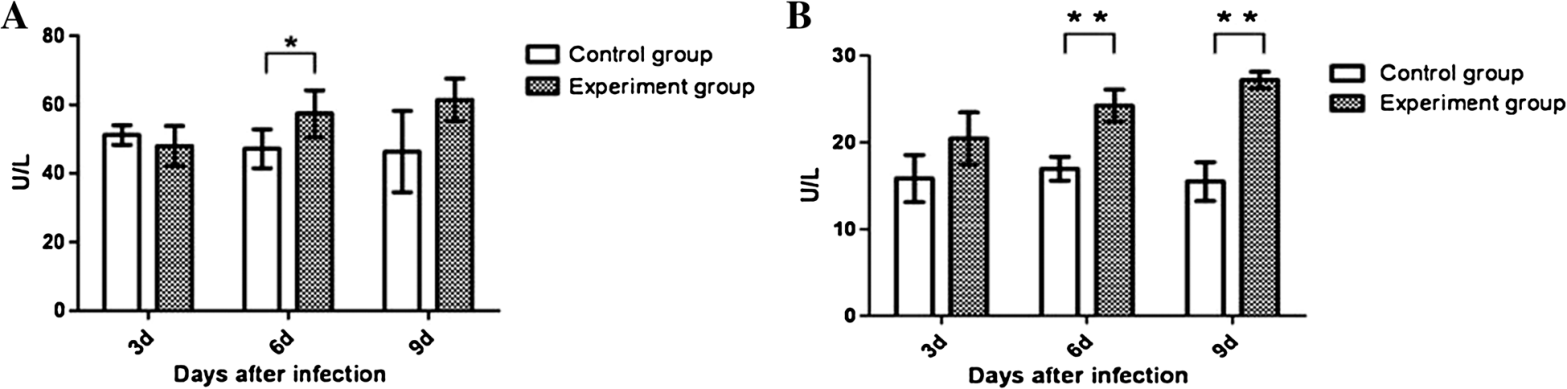
**Liver function enzymes. (A)** Alanine aminotransferase (ALT) and **(B)** aspartate aminotransferase (AST) in the serum of duck TMUV-infected mice (n = 5) infected *via* the i.c. route, on days 3, 6 and 9 post-infection. The asterisks indicate differences that are significant (* *P* < 0.05, ** *P* < 0.01).

To investigate whether the hepatic injury detected in infected mice correlates to alterations in transaminase levels, quantitative analyses of alanine aminotransferase (ALT, Figure 2A) and aspartate aminotransferase (AST, Figure 2B) were performed. In general, the levels of ALT on days 6 and 9 post-infection and the levels of AST on days 3, 6 and 9 after infection increased in most of the tested serum samples compared with control sera. The statistical analysis of the data indicated a significant difference from normal concentrations of AST on days 6 and 9 after infection (*P* < 0.01), and a significance increase of ALT levels was observed on day 6 (*P* < 0.05) after infection.

## Discussion

Flaviviruses are associated with highly pathogenic central nervous system (CNS) infections in humans and animals. A number of different factors affect the neurovirulence of the viruses, including the presence of necessary receptors in CNS cells, the ability of the virus to access target cells and the effectiveness of the immune response in clearing virus from the brain [17-20]. In our study, the pathogenicity of a non-adapted duck Tembusu-related virus originating from ducks was evaluated in BALB/c mice. Our results demonstrate that the duck TMUV-infected mice developed encephalitis, often resulting in death after i.c. inoculation. Weight loss, hind limb paralysis and blindness were strong indicators of morbidity. Inoculation *via* the i.c. route leads to productive replication of the virus in the brain. Histological changes in the brain were characterized by glial cell proliferation, formation of lymphocyte perivascular cuffing and neuronal degeneration. These changes were all reminiscent of those observed in rodent infection models with virus such as JEV [21-23], WNV [24], DENV [25] and YFV [26]. Viral antigens were detected in neural cells of the brain, and the development of clinical symptoms coincided with progressive inflammatory infiltration. Together, these data suggest that duck TMUV originating in ducks is neurotropic in a mouse model and could be important for understanding the infectious process for other zoonotic flaviviruses.

In addition to encephalitis, flavivirus infections can systematically cause hepatitis (YFV), shock syndrome (DENV) or hemorrhagic fever. The presence of viral RNA in the spleen, liver and kidneys indicates that the virus spread systematically after i.c. inoculation. However, the multiplication of the virus in periphery organs appeared to be limited, with titers below the level of detection for the plaque assay. It is necessary to investigate what mediates such systemic spread and the target cells in these visceral organs. The spread of the virus by a hematogenous route cannot be ruled out, although no viremia was detected in this study using plaque assays. The wound in the brain created by i.c. inoculation and inflammatory lesions may facilitate virus penetration by increasing blood brain barrier (BBB) permeability. Several reports have shown that breaches in the BBB induced by sham i.c. inoculation or chemical reagents play an important role in CNS invasion by JEV and variant WNVs [22,27].

In this study, mild to moderate lymphocyte depletion in the germinal centers of the spleen were observed 3–9 days after infection. An increase in splenic lymphocyte depletion occurred in parallel with inflammatory processes in the brain (Table 3). In contrast to the pathological changes in monkey and hamster models infected with YFV [28,29], no significant lymphocytic necrosis or viral antigen were found in the spleen. This result seems to support that lymphocytes were recruited to the brain rather than by direct viral cytopathic effects on splenic lymphocytes. It has been demonstrated that i.c. infection with dengue-3 virus induces encephalitis in BALB/c mice, with increased leukocyte rolling and adhesion in meningeal vessels and infiltration of immune cells to the brain [25].

In addition to these alterations, we also observed extensive steatosis in the liver on day 9 post-infection. We evaluated hepatic injury by measurements of serum transaminase levels, as with other related flavivirus infections. We observed a significant increase in the level of the enzymes ALT and AST on days 6 and 9 post-infection, respectively. The increased levels of ALT and AST are a remarkable feature of most flaviviral infectious diseases. Furthermore, in addition to liver damage, we also investigated the viral replication in the hepatic tissue of duck TMUV i.c. inoculated mice. These data suggest that duck TMUV replicates in liver tissue. In addition, fatty degeneration was also found in the kidneys in the later stage of infection. This may indicate metabolic disturbance, the same as in the liver. Similar findings have been described in human and animal models infected with DENV or YFV [30]. However, fatty degeneration in duck TMUV-infected mice is more transient, and no significant inflammatory response was observed in focal areas. The exact mechanism(s) responsible for this remain to be investigated.

## Conclusion

In summary, the results presented in this paper demonstrate that BALB/c mice can be productively infected with duck TMUV *via* i.c. inoculation. The virus exhibits a high neurovirulence, causing lesions typical of many other encephalitis flaviviruses. Splenic lymphocyte depletion, along with hepatic and renal fatty degeneration, occurred in the infected mice, making this a potential model for pathophysiological studies of flaviviruses.

## Material and methods

### Virus

We used a duck TMUV-jxsp strain isolated from the brain of infected ducks as described previously [10]. The virus was isolated by two passages in duck embryos and passaged three times in BHK-21 cells, resulting in a titer of 2.5 × 10^6^ plaque-forming units (PFU)/ml.

### Ethics statement

Animal experiments were conducted in accordance with the guidelines of the Beijing Municipality on the Review of Welfare and Ethics of Laboratory Animals and approved by the Beijing Municipality Administration Office of Laboratory Animals. Animal infection experiments were approved by the China Agricultural University Animal Ethics Committee. Mice were anesthetized with Zoletil 50 (Virbac, SanteAnimale) in phosphate-buffered saline (PBS) by subcutaneous administration immediately prior to infection.

### Animal infection

Female specific pathogen-free (SPF) BALB/c mice (three- to eight-weeks old) were purchased from Merial-Vital Laboratory Animal Technology (Beijing, China). To investigate the effective infection route, three- and six-week-old mice were infected with 7.5 × 10^4^ PFU of duck TMUV by s.c., i.p. and i.c. inoculation (n = 5). Infected mice were examined daily for 15 days for feed uptake, body weight and clinical signs. Then, three- to eight-week-old mice were infected i.c. with 7.5 × 10^4^ PFU of the virus suspension in 30 μl of PBS to compare the age difference susceptibility.

For determination of the 50% lethal end-points (LD_50_), five three- and six-week-old mice were inoculated with decimal dilution of the virus. Mice were monitored for 15 days, and the doses causing 50% mortality were calculated using the Reed and Muench method [21].

### Pathogenesis studies

To examine the efficiency of virus replication, 30 mice (six-week-old) were infected via the i.c. route with 7.5 × 10^3^ PFU of the virus (approximately 0.25 LD_50_) resuspended in 30 μl of PBS. Mice in the negative control group (n = 15) were inoculated with PBS. Five infected mice and three control mice were randomly selected for bleeding to detect viremia during the first two days of infection. On days 3, 6, 9 and 12 post-infection, five infected mice and three control mice were euthanized. Heparinized blood and tissue samples of brain, heart, lung, liver, spleen and kidneys were aseptically removed from both infected and control mice. Half of each organ was frozen at −70°C, and another portion of each organ was fixed in 10% neutral-buffered formalin. During the observation period, one mouse died on the seventh day, and two died on the eighth day, which were not included in the final data analysis.

### Histology and immunochemistry

Fixed tissue samples were embedded in paraffin, cut into 4-μm thick sections and stained with hematoxylin and eosin (HE) using standard methods. The extent and severity of the pathological changes in each tissue were evaluated and scaled according to the lesion areas. In evaluating changes in the brain, lesions were graded according to the following scales: -, none; +, 1–3 focal inflammatory infiltrates, perivascular cuffing and microglial hyperplasia per high power field (HPF); ++, lesions covering 25% of a HPF; #, lesions covering >75% of a HPF. In spleen sections, lymphoid hyperplasia, lymphoid depletion and splenic macrophage activation were used as parameters and scored in the same manner as for the brain. For the liver and kidneys, inflammatory cell infiltration and fatty degeneration were used as parameters for scoring.

For immunohistochemistry, deparaffinized sections were treated with 3% H_2_O_2_ solution for 20 min to eliminate endogenous peroxidase activity. Tissue sections were further incubated with 5% horse serum in PBS at 37°C for 60 min to block nonspecific binding sites. For detection of duck TMUV-infected cells, a monoclonal antibody against a flavivirus group antigen, HB112 (1:200; ATCC, USA), was incubated with sections at 37°C for 1 h. After washing, the sections were incubated with peroxidase-conjugated goat anti-mouse IgG (1:100; Sigma) at 37°C for 1 h. The sections were then rinsed in PBS, and an AEC staining kit (ZSGB Bio Ltd, Beijing) was used to develop the color. Following incubation at room temperature for 5 min, the tissue sections were washed and counterstained with hematoxylin.

### Plaque assays

The tissue homogenates and blood were tested for infectious virus by plaque assays utilizing BHK-21 cells following an established method [31]. Briefly, tissue samples were weighed and homogenized individually, and suspensions were centrifuged at 10 000 × *g* for 10 min at 4°C. BHK-21 cell monolayers in 24-well plates (2 × 10^6^ cells/well) were inoculated in triplicate with 0.2 ml of 10-fold serially diluted samples. After incubation at 37°C for 1 h, the inoculum was removed, and the cells were overlaid with 1 ml/well of overlay medium (1% agarose in Dulbecco’s modified Eagle’s medium supplemented with 1% fetal calf serum and antibiotics). Three days later, 0.5 ml of 1% agarose containing 0.02% neutral red was added, and the cells were incubated at 37°C in the dark for 12 h. Plaques were counted, and virus concentrations were determined and expressed as PFU/ml.

### Biochemical analysis of serum hepatic enzyme

Adult (six-week-old), female BALB/c mice were inoculated intracerebrally with 7.5 × 10^3^ PFU of the virus as in the pathogenesis study above. Five infected mice and five control mice were bled on days 3, 6 and 9 after infection (only three mice were alive on day 9 after infection), and serum samples were obtained after centrifugation at 200 *g* for 10 min and stored at −80°C. Determinations of the levels of alanine aminotransferase (ALT) and aspartate aminotransferase (AST) were performed using commercial kits (Infinity ALT, and Infinity AST; Beijing Airan Diagnostics, China), according to the manufacturer’s instructions. Student *t*-tests were performed to evaluate the differences between the control and experimental groups.

### Detection of viral RNA with Real-time PCR

Primers and TaqMan probes were designed by aligning E gene sequences of duck TMUV strains (GenBank accession no. JF312912). The primers for the detection of duck TMUV were F2 (5′-CAGTTTTCATACATGGTTCCACG-3′) and R2 (5′-CGGTAC CATAATCCTCCATCTCAGC-3′). The sequence of the TaqMan probe was 5′-FAM-AGCCCAGCAGTCGC-MGB-3′.

Total RNA was extracted from different tissue and serum samples using a TANBead Viral DNA/RNA Auto Kit (Taiwan Advanced Nanotech Inc. Taiwan) according to the manufacturer’s instructions. Reverse transcription (RT) and polymerase chain reaction (PCR) amplification were performed using a One Step PrimeScriptTM RT-PCR Kit (TaKaRa) as previously described [32]. An ABI 7500 Fast Real-Time PCR System (Applied Biosystems) was used with the following thermal cycling profile: 42°C for 5 min, initial denaturation at 95°C for 10 s and then 40 cycles of 95°C for 10 s and 60°C for 35 s.

## Competing interests

The authors have declared that no competing interests exist.

## Authors’ contributions

JS and KT designed the research; SL, XL, LZ, YW, XY and WS performed the research; JS, SL, KT and BH analyzed the data; JS and SL wrote the paper. All authors read and approved the final manuscript.
